# Effect of high-roughage backgrounding duration on carcass traits and meat quality of young Nellore bulls

**DOI:** 10.1007/s11250-026-04936-8

**Published:** 2026-02-24

**Authors:** Adailton Camêlo Costa, Cris Luana de Castro Nunes, Ana Clara Franklin Santos, Thomas Joaquim Alvarenga Lessa, Guilherme Henrique de Freitas, Pauliane Pucetti, Luciano Saraiva Santos, Nathália Veloso Trópia, Sebastião de Campos Valadares Filho, Mario Luiz Chizzotti

**Affiliations:** Department of Animal Science, Universidade Federal de Viçosa, Viçosa, MG 36570-900 Brazil

**Keywords:** Beef cattle, Meat color, Postmortem aging, Proteolysis, Tenderness

## Abstract

This study evaluated the effects of different high-roughage backgrounding durations (0, 28, 56, and 84 days) on carcass traits and meat quality of 36 young Nellore bulls (265 ± 5 kg initial body weight). After this phase, animals were finished on high-energy diets and slaughtered 154 days after the beginning of the trial. Carcass traits, meat quality, and aging effects (0, 7, and 14 days) were assessed. Data were analyzed using a completely randomized design with four treatments (backgrounding durations) and using a 4 × 3 factorial arrangement for variables evaluated across aging times. Average daily gain was greatest in non-backgrounded animals (1.12 kg/d) and lowest in those backgrounded for 84 days (0.62 kg/d). Prolonged backgrounding (84 days) reduced hot and cold carcass weights and yield, whereas no differences were observed among 0-, 28-, and 56-day groups. Most meat quality parameters, including pH, backfat, sarcomere length, exudate losses, shear force, lipid oxidation, and nitric oxide concentration, were unaffected by treatments. However, 84-day backgrounding increased meat and fat lightness (L*) and reduced meat hue angle. Aging improved myofibrillar fragmentation index (MFI) and altered water losses, lipid oxidation, nitric oxide, and color attributes. Muscle chemical composition (moisture, protein, fat, and ash) did not differ among treatments. Extending high-roughage backgrounding diets up to 56 days did not compromise performance, carcass yield, or meat quality. However, longer durations (84 days) negatively affected performance and carcass traits. Therefore, a 56-day strategy may represent a suitable option for maintaining efficiency while providing flexibility for tropical pasture-to-feedlot systems.

## Introduction

Beef cattle production is one of the pillars of the global meat supply, playing a strategic role in food security and economic stability. Brazil has an estimated commercial herd of 186.9 million head, ranking second worldwide and establishing itself as the leading exporter of beef globally (USDA 2025). In recent years, shortening the beef production cycle by reducing slaughter age and increasing the quantity and quality of the feed offered has been crucial for gaining market share and improving beef quality (Lopes et al. 2023). While conventional systems typically finish cattle at around 24 months of age, it is technically feasible to shorten the production cycle to 12–14 months using energy-dense diets. However, these intensive systems are often economically challenging, given the high cost of feed inputs and the narrower profit margins.

The use of high-roughage diets during the backgrounding phase represents an effective strategy to reduce production costs between weaning and finishing (Snider et al. 2025). When backgrounding occurs on pasture, animals may experience periods of nutritional restriction. Therefore, providing conserved forage in a feedlot system can help prevent undernutrition while reducing reliance on costly high-concentrate diets. This approach can be applied immediately after weaning, offering a practical alternative to decrease pasture pressure by maintaining animals in feedlot systems on roughage-based diets until the finishing phase. However, the optimal duration of the backgrounding phase remains poorly defined, and its effects on carcass characteristics when animals are finished under a similar feed regimen are not well established.

Sustainable intensification practices have demonstrated effectiveness in reducing slaughter age without compromising animal performance (Pereira et al. 2024; Millen and Arrigoni 2013). In addition to productive efficiency, it is essential to assess the impact of these production systems on meat quality, considering that attributes such as pH, color, tenderness, and intramuscular fat content, which are directly influenced by nutritional and management factors, particularly during the final feeding stages (Koohmaraie and Geesink 2006; Bressan et al. 2011; Aroeira et al. 2016).

Because feeding duration directly influences animal growth and body composition, we hypothesized that shorter backgrounding periods would result in lighter carcasses and reduced fat deposition, key factors affecting meat quality. Therefore, this study aimed to assess the influence of different backgrounding durations (0, 28, 56, and 84 days) with high-roughage diets on the carcass characteristics and meat quality of young Nellore bulls in a feedlot system.

## Materials and methods

This study was conducted at the experimental feedlot of the Department of Animal Science at the Federal University of Viçosa, Minas Gerais, Brazil, from August to December 2021. All procedures were previously approved by the Institutional Animal Care and Use Committee of the Federal University of Viçosa (protocol number 032/2021).

### Animals and experimental design

**Fig. 1:**
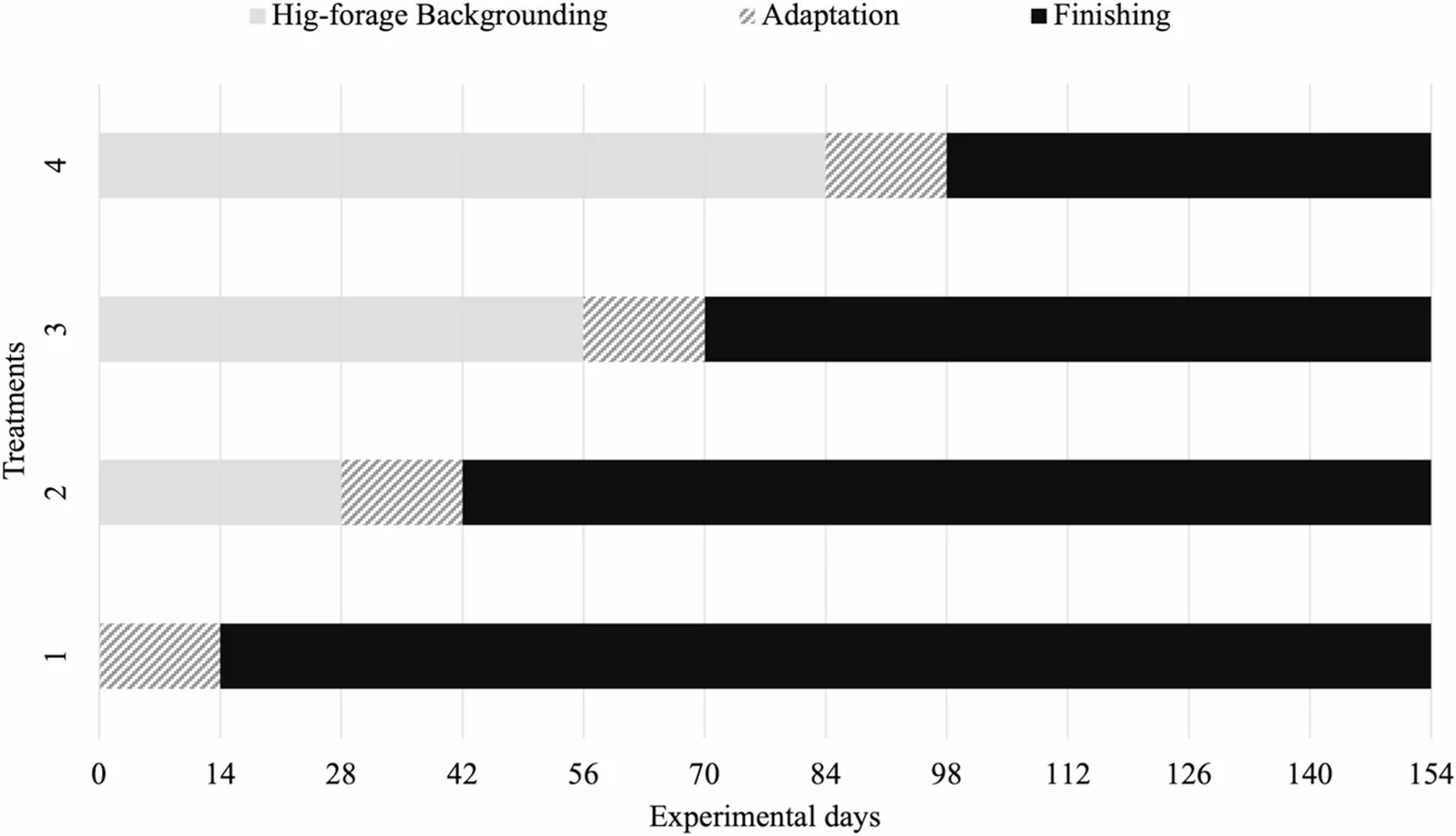
Schematic representation of the experimental period of each treatment: (1) adaptation period of 14 d + finishing phase of 140 d; (2) backgrounding phase of 28 d + adaptation period of 14 d + finishing phase of 112 d; (3) backgrounding phase of 56 d + adaptation period of 14 d + finishing phase of 84 d; and (4) backgrounding phase of 84 d + adaptation period of 14 d + finishing phase of 56 d

Thirty-six early maturing Nellore bulls with an initial body weight of 265 ± 5 kg were randomly allocated to four treatment groups (*n* = 9 per treatment). Treatments corresponded to different backgrounding durations (0, 28, 56, or 84 days) on a high-roughage diet based on sorghum silage, with a roughage-to-concentrate ratio of 80:20, followed by a 14-day adaptation to the finishing diet. Consequently, the finishing periods lasted 140, 112, 84, or 56 days, respectively (Fig. 1), totaling 154 days of trial. During the finishing phase, corn silage was used as the roughage source, with a roughage-to-concentrate ratio of 20:80. Diets were formulated according to BR-CORTE recommendations (Valadares Filho et al. 2016) to support average daily gains (ADG) of 0.6 kg/day during backgrounding and 1.2 kg/day during finishing (Table 1).

**Table 1:** Proportion of ingredients and nutrient composition of the experimental diets

Items	Diets	
	Backgrounding phase	Finishing phase
Ingredients, g/kg		
Corn Silage	-	200.0
Sorghum silage	800.0	-
Ground corn	136.6	740.4
Soybean meal	28.4	23.4
Urea	4.5	4.5
Ammonium sulfate	0.5	0.5
Virginiamycin	-	1.3
Mineral mix	30.0	30.0
Analyzed Composition, g/kg		
DM^1^	286.5	661.1
OM^3^	924.3	957.5
apNDF^4^	613.5	180.7
iNDF^5^	221.9	32.9
CP^6^	91.1	109.1
EE^7^	15.2	36.9
NFC^8^	212.6	638.8

^1^Mineral premix guarantees (per kg of DM): 190–240 g of Ca, 8.5 mg of Co (Min), 428 mg of Cu (Min),19 g of S (Min), 285 mg of Fe (Min), 10.3 g of P (Min), 21 mg of I (Min), 15 g of Mg (Min), 1285 mg of Mn (Min), 715 mg of monensin, 5.7 mg of Se (Min), 43 g of Na (Min), and 1714 mg of Zn (Min), 490 g of protein nitrogen equivalent (Max). ^2^Dry matter, ^3^Organic matter, ^4^Neutral detergent fiber corrected for ash and crude protein, ^5^Indigestible neutral detergent fiber, ^6^Crude protein, ^7^Ether extract, ^8^Non-fiber carbohydrates. In the “*Ingredients*” section, the unit g/kg represents grams of the ingredient per kilogram of dry matter in the diet. In the “*Analyzed composition*” section, the unit g/kg for dry matter represents grams of dry matter per kilogram of raw material, and for the other constituents represents grams of the constituent per kilogram of dry matter

Animals were housed in covered collective pens per treatment (48 m²), fed twice daily, with *ad libitum* access to water. Individual feed intake was recorded daily throughout the experimental period using one electronic feeder per pen (AF-1000 Master; Intergado Ltda., Contagem, Minas Gerais, Brazil), which measured intake at the animal level through RFID ear tag recognition. Body weight was recorded at the beginning and at the end of the trial to determine ADG and initial and final body weight. After 154 days of the experimental period, all animals were subjected to a 16-hour feed withdrawal, weighed, and slaughtered in accordance with Normative Instruction No. 9.013/Mapa (Brasil 2017), ensuring compliance with sanitary regulations and animal welfare guidelines.

### Carcass measurements

Animals were stunned by cerebral concussion using a captive bolt pistol, followed approximately one minute later by jugular and carotid venesection, with no electrical stimulation applied during the slaughter process. Carcasses were then divided longitudinally into right and left halves, identified, washed, and weighed to obtain hot carcass weight (HCW). The left half-carcass was used to measure initial muscle pH at 45 min postmortem, immediately before entry into the chilling room, using a portable meat pH meter (Model HI 99163 - Hanna Instruments, USA). After chilling for 24 h at 4 °C without spray chilling, cold carcass weight (CCW) and final pH were recorded. Carcass yield (%) was then calculated following the equation: *carcass yield (%) = HCW (kg) / slaughter body weight (kg) × 100.*

Additionally, subcutaneous fat thickness was measured using a digital caliper after chilling. Subsequently, the Longissimus muscle was excised between the 6th and 9th ribs, from which three samples were collected. One sample was immediately frozen (day 0), while the others were aged under refrigeration at 4 °C for 7 and 14 days, respectively.

### Objective color evaluation

For objective meat color evaluation, a steak measuring 2.54 cm in thickness from each aging time sample (0, 7, and 14 days) was thawed for 16 h at 4 °C. After thawing, steaks were removed from the vacuum-sealed packaging and allowed to bloom for 30 min at 4 °C to promote myoglobin oxygenation. Color measurements were performed using a colorimeter (HunterLab MiniScan EZ 45/0 LAV), calibrated to illuminant D65. The parameters L* (lightness), a* (redness), and b* (yellowness) were recorded on the surface of each steak according to the CIELab color scale. Five readings were taken at different points on each steak, and the average value was used for statistical analysis.

Spectral data were collected across the 400–700 nm range at 10 nm intervals. The reflectance ratio at 630 nm and 580 nm (R630/580) was used directly to assess color stability during display. Reflectance values (R) at 473, 525, 572, and 700 nm were converted to reflectance attenuation (A) using the equation: *A = log(1/R)*. The relative proportions of the three redox forms of myoglobin, metmyoglobin (MetMb), deoxymyoglobin (DeoxyMb), and oxymyoglobin (OxyMb), were calculated according to the equations provided by King et al. (2023): *%MetMb = {1.395 – [(A572 − A700) ÷ (A525 − A700)]} × 100; %DeoxyMb = {2.375 × [1 – (A473 − A700) ÷ (A525 − A700)]} × 100;* and *%OxyMb = 100 – (%MetMb + %DeoxyMb).*

The estimated values of Chroma and Hue angle were calculated using the equations provided in the Meat Color Measurement Guidelines of the American Meat Science Association (King et al., 2023): Chroma = [(a*^2^ + b*^2^*)*^*0.5*^*]* and Hue = [arctangent (b*/a*)].

### Exudate losses

To evaluate thawing loss at 0, 7, and 14 days of aging, steaks (2.54 cm thick) were weighed in the frozen state, thawed, and reweighed. For cooking loss, steaks were cooked in a water bath at 71 °C for 40 min, cooled in an ice bath for 10 min to stop cooking, and stored at 4 °C for 24 h. Steaks were then removed from the package and reweighed. Both thawing and cooking losses were expressed as a percentage of the initial weight, using the formula: *Loss (%) = [(weight before – weight after) / weight before] × 100*. In addition, total loss was determined as the difference between the frozen weight and the cooked weight, expressed as a percentage of the initial frozen weight.

### Shear force measurement

Shear force was measured using the same steaks employed for cooking loss estimation. Five cylindrical cores (1.27 cm in diameter) were obtained from each steak, cut parallel to the orientation of the muscle fibers. A V-shaped blade (angle: 60°; thickness: 1.016 mm) operating at a fixed speed of 20 cm/min was used, coupled to a TA.XT2i Texture Analyzer. The peak force (N) required to shear each core was recorded, and the average of the five measurements was used as the shear force value for the sample, representing the objective tenderness.

### Sarcomere length

Sarcomere length was estimated using the laser diffraction technique described by Cross et al. (1981). Six muscle fiber fragments were excised from each sample, placed on a glass slide, and a drop of cold sucrose solution (4 °C) was added to each fiber. A helium-neon laser (λ = 632.8 nm) was directed at the fibers, and six diffraction patterns were recorded per sample. The average distance between diffraction bands was used to calculate the sarcomere length according to the following equation: *Sarcomere length (µm) = [0.6328 x D x √(T/D)2 + 1]/T*; in which: *D* = the distance in mm between the slide holder and the location where the laser diffraction bands are collected (150 mm), and *T* = the distance in mm between the extreme bands divided by 2.

### Myofibrillar fragmentation index

The myofibrillar fragmentation index (MFI) was determined according to the method described by Culler et al. (1978), with modifications by Hopkins et al. (2004). Duplicate aliquots of 50 mg of muscle tissue, free of visible fat and connective tissue, were homogenized in 30 mL of ice-cold buffer (0.1 M KCl, 1 mM EGTA, 1 mM NaN₃, 1 mM MgCl₂, and 20 mM phosphate buffer at pH 7.0, 4 °C) using an Ultra-Turrax homogenizer at 19,000 rpm for two 30 s intervals, while keeping the samples on ice. The homogenate was filtered through a 1 mm² mesh screen and centrifuged at 1,000 × g for 10 min at 2 °C. This centrifugation step was repeated three times, with resuspension of the pellet in fresh buffer after each step. Protein concentration was adjusted to 0.5 mg/mL using the biuret method (Gornall et al. 1949). Absorbance was measured at 540 nm, and the MFI was calculated by multiplying the mean absorbance value by 150.

### Chemical composition

To determine meat chemical composition, samples of the *Longissimus thoracis* muscle (approximately 90 g), free of subcutaneous fat, were finely chopped, freeze-dried, and ground. Moisture, crude protein, and ash contents were determined according to the procedures described by Silva and Queiroz (2002). The intramuscular fat content was determined in duplicate by petroleum ether extraction, using Ankom XT4 filter bags and the Ankom XT15 fat extractor (ANKOM Technology, Macedon, NY, USA), following the manufacturer’s instructions.

### Lipid oxidation

Lipid oxidation was assessed using homogenized samples (3 g/10 mL of tissue) to determine the concentrations of malondialdehyde (MDA) and nitric oxide (NO). Sample preparation was conducted according to the method described by Walsh et al. (1993). Tissue lipid peroxidation was evaluated by measuring MDA concentrations using the thiobarbituric acid reactive substances (TBARS) assay, following the methodology of Buege and Aust (1978).

Nitric oxide (NO) levels were quantified indirectly through the Griess reaction, as described by Tsikas (2007).

Protein concentrations were estimated using the method described by Bradford (1976), and the results for MDA and NO were expressed as µmol and µM equivalents per gram of soluble protein, respectively.

### Statistical analysis

The experiment was performed in a completely randomized design with four treatments (0, 28, 56, and 84 days of backgrounding diet) and nine replicates per treatment. Data normality was assessed using the Shapiro–Wilk test. Statistical analyses were performed using the GLM procedure of SAS (SAS Institute Inc., Cary, NC, USA), using the following model: *Y*_*ij*_ *= µ + T*_*i*_ *+ ε*_*ij*_; in which *Y*_*ij*_ is the response variable, *µ* is the overall mean, *T*_*i*_ is the fixed effect of treatment (*i* = 1 to 4), and *εij* is the random error, assumed to be normally distributed with homogeneous variance.

For variables evaluated over aging time, a 4 × 3 factorial arrangement (four treatments × three aging times: 0, 7, and 14 days) was employed. Therefore, the following model was applied: *Y*_*ijk*_ *= µ + T*_*i*_ *+ A*_*j*_ *+ (T × A)*_*ij*_ *+ ε*_*ijk*_; in which *Y*_*ijk*_ is the observed value of the dependent variable in animal *k*, treatment *i*, and aging time *j*, *µ* is the overall mean, *T*_*i*_ is the fixed effect of treatment (*i* = 1 to 4), *Aj* is the fixed effect of aging time (*j* = 0, 7, and 14 days), (*T × A*) _ij_ is the interaction between treatment and aging time, and *εij* is the random error.

When significant effects were detected (*P* ≤ 0.05) for the main factors or their interaction, means were compared using Tukey’s test.

## Results

The ADG decreased as backgrounding duration increased (*P* < 0.001), with the highest value observed in animals not subjected to backgrounding (1.12 kg/d) and the lowest in those backgrounded for 84 days (0.62 kg/d), as shown in Table 2. Average final body weight tended to be lower in animals backgrounded for 84 days, averaging 361.39 ± 15.6 kg, compared with those in the other groups, which averaged 422.83 ± 8.7 kg (*P* = 0.053). Similarly, hot and cold carcass weights were reduced (*P* < 0.001) in animals subjected to 84 days of backgrounding, while no differences were detected among the 0-, 28-, and 56-day groups. Carcass yield was also lower (*P* = 0.020) in animals backgrounded for 84 days relative to the shorter phases. Neither initial nor ultimate pH was affected by treatments (*P* ≥ 0.670). Also, backfat thickness and sarcomere length did not differ among groups (*P* ≥ 0.216).

**Table 2:** Performance and carcass traits of Nellore cattle subjected to different high-forage backgrounding durations

Item	Backgrounding phase (days)				SEM^1^	*P*- value
	0	28	56	84		
Average daily gain, kg	1.12^a^	0.97^b^	0.91^b^	0.62^c^	0.53	< 0.001
Initial body weight, kg	265.78	264.67	265.72	263.83	9.95	0.999
Final body weight, kg	442.56^a^	417.44^a^	408.5^a^	361.39^b^	15.00	0.053
Hot carcass weight, kg	274.57^a^	257.64^a^	249.32^a^	216.77^b^	9.07	< 0.001
Cold carcass weight, kg	271.44^a^	254.67^a^	246.83^a^	214.21^b^	8.99	< 0.001
Initial pH	6.68	6.67	6.71	6.75	0.07	0.875
Ultimate pH	5.76	5.82	5.92	5.78	0.10	0.670
Carcass yield, %	61.35^a^	61.00^a^	60.40^a^	59.37^b^	0.45	0.020
Backfat thickness, mm	4.44	3.10	3.79	2.47	0.68	0.216
Sarcomere, µm	1.28	1.29	1.19	1.32	0.07	0.631

¹Standard error of the mean. ^a, b^ Means with different superscripts in the same row differ significantly (*P* < 0.05)

Thawing and cooking losses were not affected by backgrounding duration (*P* > 0.05) but were influenced by aging time (Table 3). Thawing loss was greater at 7 days of aging (7.94%) than at 0 (6.65%) and 14 days (6.08%; *P* = 0.001). Cooking loss was higher at 7 days (15.45%) than at 0 days (14.05%), whereas values at 14 days (14.41%) did not differ from the other aging times (*P* = 0.041). Consequently, total losses followed a similar pattern, with greater values at 7 days compared with 0 and 14 days of aging (*P* = 0.001).

**Table 3:** Exudate losses, shear force, myofibrillar fragmentation index, and oxidative parameters of the *Longissimus thoracis* muscle from Nellore steers subjected to different durations of backgrounding with high-forage diets

Item	Backgrounding phase (days)				SEM^1^	Aging time (days)			SEM^1^	*P*- value		
	0	28	56	84		0	7	14		BP^2^	AT^3^	BP*AT
Thawing loss, %	6.750	6.836	6.200	7.766	0.402	6.648^b^	7.935^a^	6.080^b^	0.348	0.0559	0.0010	0.4503
Cooking loss, %	15.064	14.445	14.002	15.034	0.463	14.048^b^	15.454^a^	14.407^ab^	0.401	0.3094	0.0405	0.8684
Total losses, %	21.814	21.281	20.202	22.800	0.698	20.696^b^	23.389^a^	20.487^b^	0.604	0.0720	0.0013	0.8063
Shear force, Kgf	5.217	4.550	4.865	4.677	0.214	4.897	5.090	4.455	0.185	0.1440	0.0724	0.9812
MFI^4^	20.726^A^	22.035^A^	19.69^B^	22.187^A^	0.656	21.121^b^	23.035^a^	19.323^c^	0.568	0.0260	< 0.0001	0.0040
MDA^5^, µmol/g	0.161	0.169	0.152	0.156	0.091	0.136^c^	0.153^b^	0.183^a^	0.003	0.0828	< 0.0001	0.1216
NO^6^, µM/g	3.440	2.766	3.371	3.413	0.235	1.274^c^	3.216^b^	5.253^a^	0.203	0.1392	< 0.0001	0.3558

¹Standard error of the mean, ^2^Backgrounding phase, ^3^Aging time, ^4^Myofibrillar fragmentation index, ^5^Malondialdehyde, ^6^Nitric oxide

^a, b,c^ Means with different lowercase superscripts in the same row differ significantly (*P* < 0.05) among aging time. ^A, B^ Means with different uppercase superscripts in the same row differ significantly among backgrounding treatments (*P* < 0.05)

**Fig. 2:**
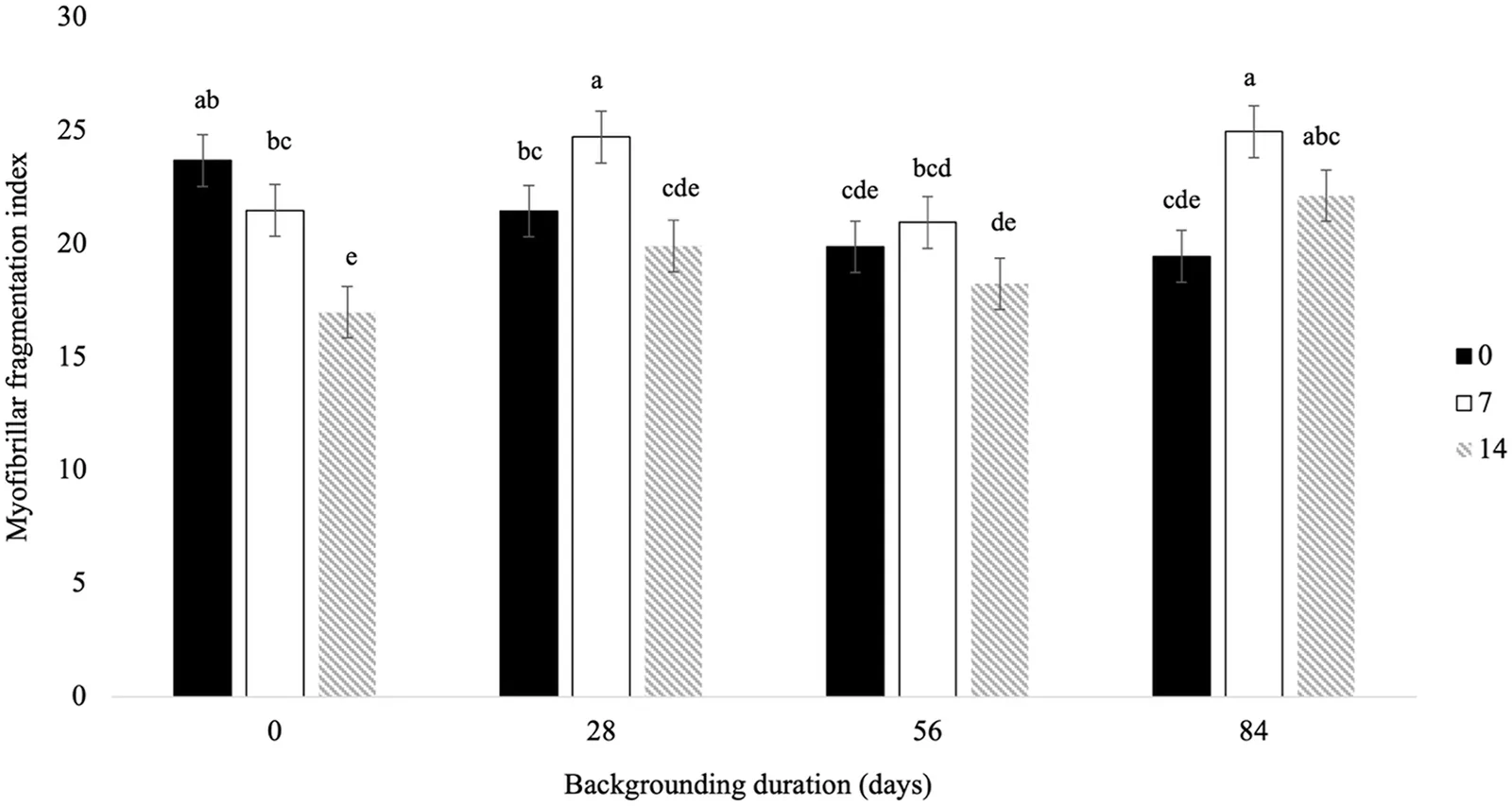
Myofibrillar fragmentation index from young Nellore bulls subjected to 0, 28, 56, or 84 days of backgrounding phase and aged for 0, 7, or 14 days ^a, b,c, d,e^ Means with different lowercase superscripts differ significantly (*P* < 0.05)

Shear force was not affected by backgrounding or aging (*P* ≥ 0.072). However, backgrounding for 56 days resulted in lower MFI values compared with the other treatments, whereas aging for 14 days markedly increased MFI relative to 0 and 7 days. Moreover, a significant interaction between backgrounding and aging was detected for MFI (*P* = 0.004). At 7 days of aging, MFI was higher in carcasses from animals backgrounded for 28 and 84 days compared with 0 and 56 days. In contrast, at 0 and 14 days of aging, no consistent differences were observed among backgrounding durations (Fig. 2).

Lipid oxidation, expressed as malondialdehyde (MDA) concentration, was not affected by backgrounding (*P* = 0.083) but increased with aging time (*P* < 0.001). Similarly, nitric oxide (NO) concentration was unaffected by backgrounding (*P* = 0.139) but increased with aging (*P* < 0.001).

Meat color was affected by both backgrounding duration and aging time (Table 4). The L* value increased (*P* < 0.001) in animals backgrounded for 84 days compared with the other groups and increased with aging (*P* < 0.001), with higher values at 7 and 14 days relative to day 0. The a*, b*, and chroma values were not affected by backgrounding duration (*P* = 0.0713; *P* = 0.2362; and *P* = 0.9169, respectively). However, a* parameter decreased with aging (*P* < 0.001), showing higher values at day 0 than at 7 and 14 days. The b* value decreased at 7 days compared with 0 and 14 days of aging (*P* = 0.0003). Chroma was lower at 7 days, followed by 14 and 0 days of aging (*P* < 0.001). Hue angle was reduced in animals backgrounded for 84 days (*P* = 0.0014) and decreased with aging (*P* = 0.0003). Metmyoglobin proportion was not influenced by backgrounding duration (*P* = 0.200) but was higher after 7 (39.22%) and 14 days (41.20%) compared with 0 days (34.18%; *P* = 0.019). The proportion of deoxymyoglobin and oxymyoglobin was not affected by backgrounding (*P* ≥ 0.199) or aging (*P* ≥ 0.064).

**Table 4:** Color parameters of the *Longissimus thoracis* muscle from Nellore steers subjected to different backgrounding with high-forage diets

Item	Backgrounding phase (days)				SEM^1^	Aging time (days)			SEM^1^	*P*- value		
	0	28	56	84		0	7	14		BP^2^	AT^3^	BP*AT
**Meat color**												
L*	36.157^B^	37.459^B^	37.578^B^	40.033^A^	0.627	35.796^b^	38.412^a^	39.212^ab^	0.543	0.0004	< 0.0001	0.9908
a*	14.235	14.128	14.46	13.409	0.292	15.452^a^	13.111^b^	13.61^b^	0.253	0.0713	< 0.0001	0.8193
b*	13.102	13.409	13.22	13.879	0.285	13.847^a^	12.554^b^	13.806^a^	0.247	0.2362	0.0003	0.8173
Chroma	19.384	19.517	19.641	19.336	0.334	20.777^a^	18.188^c^	19.443^b^	0.289	0.9169	< 0.0001	0.6496
Hue	47.441^A^	46.404^A^	47.494^A^	43.942^B^	0.704	48.148^a^	46.27^b^	44.543^c^	0.610	0.0014	0.0003	0.998
%MetMb	39.579	40.0891	34.415	38.723	2.059	34.183^b^	39.217^a^	41.204^a^	1.783	0.1997	0.0192	0.8809
%DeoxyMb	13.667	13.565	13.951	13.853	0.667	14.742	13.597	12.939	0.577	0.9763	0.0872	0.651
%OxyMb	46.457	46.345	51.535	47.349	1.9497	51.073	47.111	45.58	1.689	0.1994	0.0646	0.8387
**Fat color**												
L*	68.294^B^	68.253^B^	68.851^B^	70.298^A^	0.553	70.316^a^	68.78^b^	67.676^b^	0.479	0.0348	0.0008	0.3723
a*	4.196	4.066	3.96	3.584	0.211	3.422^a^	3.531^a^	4.902^b^	0.183	0.2056	< 0.0001	0.930
b*	15.409	14.552	14.898	15.071	0.292	14.939	14.804	15.205	0.253	0.2206	0.5242	0.9525
Chroma	16.203	15.45	15.467	15.3674	0.329	15.32	15.493	16.052	0.284	0.5452	0.1699	0.9904
Hue	15.092	15.675	14.598	13.19	0.695	12.55^b^	13.377^b^	17.99^a^	0.602	0.0788	< 0.0001	0.5108

¹Standard error of the mean; ^2^Backgrounding phase; ^3^Aging time. L* – lightness; a* – redness; b* – yellowness; MetMb – metmyoglobin; DeoxyMb – deoxymyoglobin; OxyMb – oxymyoglobin

^a, b,c^ Means within a row with different lowercase superscript letters differ significantly (*P* < 0.05) among aging time. ^A, B^ Means within a row with different uppercase superscript letters differ significantly (*P* < 0.05) among backgrounding treatments

Regarding fat color, L* was greater in carcasses from animals backgrounded for 84 days (*P* = 0.035) and decreased with aging (*P* = 0.001), with a greater value at day 0 compared with 7 and 14 days. Fat a* was not affected by backgrounding (*P* = 0.206) but increased at 14 days of aging (*P* < 0.001) compared to 0 and 7 days. Fat b* and chroma were not influenced by either factor (*P* ≥ 0.170). The fat hue angle was unaffected by backgrounding (*P* = 0.079) but increased (*P* < 0.001) with aging, with higher values at day 14 compared with days 0 and 7.

The chemical composition of the *Longissimus thoracis* muscle (Table 5) was not affected by backgrounding duration (*P* ≥ 0.225). Moisture, protein, intramuscular fat, and ash contents remained similar among treatments, averaging 72.3%, 20.6%, 4.7%, and 1.0%, respectively.

**Table 5:** Chemical composition of the *Longissimus thoracis* muscle from Nellore steers subjected to different high-forage backgrounding durations

Item	Backgrounding phase (days)				SEM^1^	*P*- value
	0	28	56	84		
Moisture, %	72.1662	72.1416	72.3318	72.594	0.5322	0.9199
Protein, %	21.1555	20.6033	20.2155	20.5633	0.3138	0.2247
Intramuscular fat, %	4.4366	4.8444	4.8677	4.5177	0.3822	0.7997
Ash, %	1.0377	1.0188	1.0288	1.0255	0.0173	0.8916

## Discussion

In the present study, extending the backgrounding period to 84 days with a high-forage diet reduced ADG from 1.12 to 0.62 kg, final body weight from 442.56 to 361.39 kg, HCW from 274.57 to 216.77 kg, CCW from 271.44 to 214.21 kg, and carcass yield from 61.35 to 59.37%. These results are consistent with previous reports (Shibata et al. 2019; Reuter and Beck 2013; Ku et al. 2021) and can be explained by the lower energy density of forage-based diets during the growing phase, which limited animal performance even under *ad libitum* intake, combined with insufficient time for compensatory growth during the finishing phase. Although compensatory gain is well documented in cattle subjected to moderate feed restriction (Galyean and Hales 2023; Keogh et al. 2015), its benefits are more evident when animals are provided with both adequate time and high-energy diets during the realimentation phase. In this study, the relatively short finishing period for steers backgrounded for 84 days likely restricted their ability to express compensatory growth fully.

Conversely, steers backgrounded for up to 56 days maintained comparable carcass characteristics to those not backgrounded, as reflected in similar final body weights, carcass weights, and yields. This suggests that limiting the high-forage backgrounding phase to ≤ 56 days may help reduce feeding costs without compromising carcass performance. Menezes et al. (2019) reported that cattle subjected to different growing strategies can produce comparable carcasses when finished to a common endpoint.

Ultimate pH values remained slightly above the normal range of 5.4–5.7 (Braden 2013). According to Meat Standards Australia, an ultimate pH of 5.7 represents the upper limit for acceptable beef quality, as higher values are associated with darker meat color and reduced eating quality (MLA 2011). In addition, the sarcomere length values observed in this study suggest a potential occurrence of cold shortening, an irreversible contraction of muscle fibers when rapidly chilled, as values below 1.8 μm are considered indicative of cold-induced shortening (Ertbjerg and Puolanne 2017). Shear force values ranged from 4.45 to 5.21 kgf across backgrounding durations and aging times. In this context, Miller et al. (2001) reported that shear force values around 4.3 kgf are associated with 86% consumer acceptance, whereas values above 4.9 kgf reduce acceptance to approximately 25%. Although shear force did not differ among backgrounding periods or aging times (Table 3), the relatively elevated values observed may have been influenced by the potential occurrence of cold shortening.

The results observed for thawing and cooking losses after a 7-day aging period can be attributed to the structural modifications that occur in the muscle matrix during meat aging. The action of proteolytic enzymes promotes the degradation of structural proteins, resulting in a reduction of water-holding capacity (Pearce et al. 2011; Huff-Lonergan and Lonergan 2005). In the present study, thawing loss across aging times ranged from 6.08 to 7.94%, values that are consistent with reports indicating that approximately 5% of exudate is lost during aging periods of 3 to 8 days, with losses increasing to up to 9% during aging periods of 14 to 21 days (Purslow et al. 2016). Losses during these processes are inevitable due to the presence of free water within the intercellular space of muscle tissues, which is released during thawing and cooking (Rahman et al. 2014).

Lipid oxidation increases with aging time, as described in the literature (Lee et al. 2021; Ismail et al. 2008). The values obtained in this study are within the acceptable limits for meat oxidation, as reported by Spaziani et al. (2011) and Gray et al. (1996), where rancidity indicators are above 1.5 mg/kg, and consumer-perceptible levels are around 0.5 mg/kg. These are positive indicators, suggesting that both the backgrounding duration and aging time were not detrimental to meat quality.

The MFI reflects the extent of myofibrillar degradation and is generally positively correlated with meat tenderness (Veiseth et al. 2001). In the present study, however, changes in MFI were not sufficient to produce differences in shear force across treatments. At 0 and 7 days of aging, a noticeable increase in MFI was observed, which is consistent with the activity of calpains, enzymes responsible for post-mortem myofibrillar degradation. Conversely, the reduction in MFI at 14 days of aging represents a trend contrary to expectations, as continued fragmentation would normally be anticipated. This response may be explained by the increased nitric oxide levels observed at 14 days, which may inhibit calpain activity and the degradation of structural proteins, such as desmin, thereby reducing myofibrillar fragmentation (Marino et al. 2015).

The observed interaction for MFI indicates that backgrounding duration is not an isolated factor but interacts dynamically with the post-mortem aging process (Fig. 2), modulating the extent of myofibrillar degradation. Similar responses have been reported in other animal models, in which feed restriction influenced MFI and muscle proteolysis by reducing the rate and extent of post-mortem myofibrillar fragmentation compared with animals fed *ad libitum*, possibly due to alterations in calpain system activity and calpastatin levels (Leonardo et al. 2008).

Meat color is an important factor in consumer perception of quality. Beef from forage-fed cattle tends to exhibit fat with a yellowish or orange hue due to the presence of carotenoids in pastures or diets rich in roughage. This coloration is often negatively perceived at the point of sale, as consumers are used to the bright red color typical of meat from animals fed grain-based diets (Duckett et al. 2013). According to Holman and Hopkins (2021), acceptance thresholds for beef color have been compiled from various studies, indicating that color is considered acceptable when L* > 31.4; a* > 14.5; b* > 6.3; Hue > 22.5; and Chroma > 17.4. In this study, color parameters remained within acceptable ranges, except for the a* value, which was slightly below the recommended threshold across backgrounding phases, averaging 14.05, and at 7 and 14 days of aging, averaging 13.11 and 13.61, respectively. Although meat quality preferences vary among countries, lower a* values are associated with a reduced probability of consumer purchase, as decreases in redness negatively affect visual appeal and market value (Witberler et al. 2025).

During the aging process, it is common to observe an increase in meat L*, as seen in this study. Similar results were reported by Wicks et al. (2024), who observed increased lightness in meat from pasture-fed animals compared to those finished on grain-based systems. This pattern may positively indicate that aging can contribute to the visual improvement of meat from cattle backgrounded or finished on high-roughage diets. In the present study, the values of a*, b*, chroma, and hue were reduced during aging. Mateus et al. (2018) found similar reductions in these parameters over the aging period. This phenomenon can be explained by biochemical changes in myoglobin, the primary pigment responsible for meat color. As aging progresses, myoglobin oxidation leads to the formation of metmyoglobin and the consequent reduction in meat color intensity (King et al. 2023).

An increase in subcutaneous fat lightness was observed in this evaluation in the meat of animals subjected to longer backgrounding periods. This result may be related to the dilution of carotenoids deposited in tissues during the feeding phase with diets rich in roughage. Although the initial diet has the potential to intensify the yellowish coloration of fat, the subsequent lipid deposition during the finishing phase with concentrate may have reduced the concentration of these pigments, promoting a lighter appearance of the fat (Dunne et al. 2009).

The a* value of fat increased with aging time, possibly due to lipid oxidation reactions and interactions between residual pigments (Wood et al. 2008). Similarly, hue values increased over the aging period, which can be explained by oxidative processes occurring during vacuum aging of fat, promoting color changes toward more yellowish and brownish tones (Mancini and Hunt 2005).

Regarding the chemical composition of the meat, moisture, protein, intramuscular fat, and ash contents remained statistically similar among treatments, indicating that high-forage backgrounding for up to 84 days did not influence the proximate composition of the meat. Ether extract content, indicative of intramuscular fat, was not greater even in animals subjected to longer finishing periods. Although diets with higher concentrate inclusion may favor greater marbling deposition (Pethick et al. 2004), this effect was not observed in the present study. Similar to our results, the meat proximate composition of Nellore cattle typically includes about 75% moisture, 19–25% protein, less than 5% intramuscular fat, and approximately 1.0% ash, reflecting the lean phenotype of this *Bos indicus* breed (Ferreira et al., 2020). Consequently, feeding strategies, particularly high-energy diets, tend to primarily affect fat deposition rather than other components of meat composition.

## Conclusion

In a production system of young Nellore bulls, extending the backgrounding phase with high-roughage diets for up to 56 days did not affect animal performance, carcass weight, or carcass yield. Among meat quality parameters, MFI and the color parameters L* and hue were affected, but their values remained within normal ranges. Despite the backgrounding duration, aging time improved MFI, which is positively correlated with meat tenderness. Therefore, the adoption of a 56-day high-forage backgrounding strategy represents a practical management option that balances productive performance, carcass traits, and meat quality, while offering potential feed cost savings and sufficient finishing time to allow compensatory gains.

## Data Availability

The data that support the findings of this study are available from the corresponding author upon reasonable request.
